# Neonatal Hyperthyroidism Associated with Isolated Submandibular Sialadenitis: Is it Just a Coincidence?

**DOI:** 10.4274/jcrpe.v2i1.43

**Published:** 2010-12-08

**Authors:** Ayşın Uçkun Kitapçı, Ali Süha Çalıkoğlu

**Affiliations:** 1 Bayındır Hospital, Pediatric Endocrinology, Ankara, Turkey; 2 North Carolina Childrens Hospital, Pediatric Endocrinology, Chapel Hill, USA; +90 312 287 90 00/7590-7591uckuna@yahoo.comBayındır Hospital, Pediatric Endocrinology, Ankara, Turkey

**Keywords:** Hyperthyroidism, isolated, neonatal, sialadenitis, submandibular

## Abstract

Isolated submandibular sialadenitis is extremely rare in the neonatal period. All reported cases had predisposing factors such as prematurity, dehydration, anatomic deformities, calculi, and long-term oro-gastric tube feeding. Here, we present a case of neonatal isolated submandibular sialadenitis without any known risk factors and who was diagnosed later with hyperthyroidism secondary to maternal Graves’ disease. Both sialadenitis and hyperthyroidism resolved with appropriate treatment. While both conditions are rare and important in neonatal emergency care, the possibility of neonatal hyperthyroidism should be explored in cases with isolated sialadenitis if there is no other risk factor. Possible mechanisms for this uncommon association are discussed.

**Conflict of interest:**None declared.

## INTRODUCTION

Suppurative disease of the salivary glands is uncommon in childhood. The parotid gland is more commonly involved and is usually followed by submandibular sialadenitis (1). Isolated neonatal submandibular sialadenitis is very rare and seen mostly in premature infants. Predisposing factors include prematurity, dehydration, anatomic abnormalities of the oral cavity, calculi in salivary ducts, hematogenous spread of infection from the mother, long-term oro-gastric tube feeding, and infection acquired by ingestion of contaminated formula (1).

Neonatal hyperthyroidism is also rare and mostly secondary to maternal Graves' disease. The clinical findings include irritability, poor weight gain despite good appetite, tachycardia, goiter and exophtalmus. Cardiomyopathy, congestive cardiac failure, craniosynostosis and accelerated bone maturation may develop if treatment is delayed or suboptimal (2). Several congenital abnormalities, including cardiac abnormalities, inguinal hernia, imperforate anus, have been reported in association with untreated maternal hyperthyroidism. However, sialadenitis in association with neonatal hyperthyroidism has not been reported (3). Here, we present a full-term newborn with hyperthyroidism who developed isolated submandibular sialadenitis in the first two weeks of life.

## CASE REPORT

A male infant was born via spontaneous vaginal delivery at term. His birth weight was 3147 g. His mother was diagnosed to have Graves’ disease prior to this pregnancy and was treated with propylthiouracil (PTU) throughout pregnancy. While labor and delivery were uncomplicated, mild dyspnea, attributed to laryngomalacia, was noted in the early postnatal period. At age 14 days, a swelling developed in the left submandibular region. The mass was firm and tender. The diagnosis of sialadenitis was made on clinical grounds, and confirmed by CT scan that showed an enlarged left submandibular gland with no calcification, but with round areas of low attenuation which were likely to represent a dilated submandibular duct. White blood cell count was 23 000/mm^3^ with 51% neutrophils, 2% bands, 26% lymphocytes, 10% monocytes, 1% eosinophils. The infant was admitted to a local hospital with a diagnosis of submandibular sialoadenitis and was treated with IV antibiotics (ceftriaxone 150 mg q12h). The submandibular mass showed a significant decrease in size in the first two days of admission. The patient remained afebrile throughout his hospital course and was discharged on oral amoxicillin, which was continued for 7 more days. His blood cultures were negative. 

During this outside hospital follow-up the thyroid gland was noted to be diffusely enlarged in his CT scan (obtained for sialadenitis) and thyroid function tests were obtained. Initial serum thyroxine (T_4_) level was >21 mg/dL (normal 7.0-13.5 mg/dL for newborns) and thyroid stimulating hormone (TSH) was undetectable (normal 0.45–5.50 mIU/mL).

The patient was 45 days old when referred to our center. His mother reported that he had jitteriness, difficulty in sleeping and poor weight gain despite a voracious appetite. She also noted that he had been sleeping with eyes open and having about 10 bowel movements a day. On examination he was thin, alert and irritable, moderately dehydrated. His weight was 3920 g (a weight gain of 17 g/day since birth). His pulse rate was 182/min. He had a staring look and mild proptosis. The thyroid gland was diffusely enlarged. Examination of other systems did notreveal any noteworthy findings. 

Thyroid function tests at presentation showed a T_4_ level of >24.9 mg/dL (normal 7-13.5) and an undetectable TSH level (normal 0.45-5.5 mIU/mL). Thyroid stimulating immunoglobulin index was 6.2 (normal <1.9). The diagnosis of neonatal hyperthyroidism was made and the patient was started on PTU 7.5 mg/kg/day and propranolol 2 mg/kg/day. He also received Lugol's solution (1 drop q8h on the first day of treatment) because of high risk for congestive heart failure. A gradual but steady improvement was noted in the subsequent days. After discharge, antithyroid treatment was monitored by frequent clinical and biochemical assessments and was discontinued at age nine months.

## DISCUSSION

Isolated submandibular sialadenitis is a rare condition in the newborn period. It is reported to occur as a late-onset neonatal infection at a median age of 12 days (1). The etiology is unclear, but it is speculated that reduced saliva flow due to anatomic abnormalities, calculi or dehydration play a role. A local source for infection such as long-term oro-gastric tube feeding, infection by ingestion of contaminated formula or hematogenous spread of infection from the mother are also considered as possible etiological factors (4). Prematurity has been reported as the main risk factor, suggesting that the immaturity of the immune system is also an important factor for this condition (5, 6). No risk factors were present in our patient, except for hyperthyroidism. Because there are some clinical conditions in which both thyroid and salivary glands are affected, it is plausible to argue that the association of submandibular sialadenitis and neonatal hyperthyroidism may be more than a simple coincidence. 

Although rare, reports on salivary gland involvement in patients with autoimmune thyroiditis or thyroid involvement in Sjogren’s syndrome suggest that common mechanisms may be operative in the development of thyroid and salivary gland immune disease (7). It has been reported that both glands share antigens, which could be responsible for the association between Sjogren’s syndrome and autoimmune thyroiditis (8). However, our patient's age, his clinical presentation and his good response to systemic antibiotic treatment, are findings which suggest that an acute suppurative infection, rather than a chronic inflammatory sialadenitis, may have had a role in the etiology of the sialadenitis.

The next possibility is that hyperthyroidism and/or the autoantibodies causing Graves’ disease alter saliva flow and/or content, which in turn increase the risk for sialadenitis. It is known that hyperthyroidism may influence the salivary gland functions. Koczor-Rozmus et al (9) reported up to 90% reduction in stimulated saliva volume. Saliva content is also altered by hyperthyroidism. Ford et al (10) found that the concentrations of total protein, calcium and lactate dehydrogenase activity significantly decreased in hyperthyroid patients compared to the control group, and improved following radioactive iodine treatment. Toft et al (11) demonstrated that the inability to secrete the water-soluble glycoprotein form of the ABO blood group antigens into saliva was significantly more common in patients with Graves' disease. This defect in secretion of ABO blood group antigens is associated with increased susceptibility to infection and to asymptomatic carriage of some microorganisms. It is therefore conceivable that the changes in saliva content could increase the vulnerability to infection in patients with hyperthyroidism.

The mechanism for the effects of hyperthyroidism on salivary content is not known. The salivary glands have the capacity to uptake and concentrate iodide selectively and sialadenitis is the most frequent complication of 131I therapy for thyroid cancer (12). The iodine uptake is likely due to the expression of a highly specialized iodide transporter, sodium iodide symporter (NIS) in the salivary glands. NIS is a membrane protein that mediates active I- transport in several tissues, including salivary glands as well as the thyroid gland, the stomach, the thymus and breast tissues (13). Thyrotropin receptor antibodies were shown to be present in salivary glands in relatively higher concentrations than the serum concentrations in patients with autoimmune thyroid disease (14). It is possible that these antibodies increase the iodide concentration in salivary glands by altering NIS expression, which, in turn, affects the amount or composition of the saliva. 

Finally, dehydration has been cited as a predisposing factor in previously reported cases with submandibular sialadenitis. Our patient was moderately dehydrated at presentation. Subclinical dehydration may have developed in the early neonatal period in our patient due to frequent stools and may have led to a predisposition to stasis in the salivary ducts. 

In conclusion, both sialadenitis and hyperthyroidism are rare, but dangerous conditions for newborns. Hyperthyroidism alters saliva composition, may cause dehydration and can be a contributing factor in sialadenitis. It is known that, despite their limited ability to develop an immune response and their consequent susceptibility to some infections, neonates have a relatively efficient immune system to protect them from some immune mediated disorders and autoimmune syndromes. Hyperthyroidism is one of those syndromes (15). The possible role of hyperthyroidism in neonatal sialadenitis was discussed in this presentation. This causal relationship needs to be further studied. Meanwhile, this relationship should be considered when a newborn with hyperthyroidism develops an unexplained fever and/or submandibular mass.
